# Carriage of multiple resistance genes by community and hospital isolates of *Stenotrophomonas maltophilia* resistant to the first-line drug of choice in Nigeria

**DOI:** 10.11604/pamj.2024.49.125.21124

**Published:** 2024-12-18

**Authors:** David Olusoga Ogbolu, Oyebode Armstrong Terry Alli, Adeolu Sunday Oluremi, Deborah Iyanu Ojebode, Yetunde Temilola Ogunjimi, Mark Alexander Webber

**Affiliations:** 1 Department of Biomedical Sciences, Ladoke Akintola University of Technology, Osogbo Campus, Ogbomoso, Nigeria,; 2 Antimicrobials Research Group, Institute for Microbiology and Infection, School of Immunity and Infection, University of Birmingham, Edgbaston, B15 2TT, Birmingham, United Kingdom,; 3 The Quadram Institute, Norwich Research Park, Colney, NR4 7UQ, Norwich, United Kingdom,; 4 Norwich Medical School, Norwich Research Park, Colney, NR4 7UA, Norwich, United Kingdom

**Keywords:** Stenotrophomonas maltophilia, carriers, antibiotic resistance, genomics, sequencing

## Abstract

Stenotrophomonas maltophilia (S. maltophilia) has emerged as an important nosocomial pathogen and causes life-threatening infections among the vulnerable, and occasionally in immunocompetent individuals. We examined the mechanisms of multidrug resistance in S. maltophilia isolated from sick and healthy individuals in Nigeria. Susceptibility testing and genome sequencing revealed multidrug resistance and carriage of multiple antimicrobial resistance genes in all isolates. Genes encoding resistance to fluoroquinolones, beta-lactams, aminoglycosides, chloramphenicol, sulphonamide and trimethoprim including oqxB, bla1_3, aph(3), cmlB1, sul and dfrA, variants were found in all the isolates. Genome analysis revealed multiple plasmids were present in the strains including ColBS512, ColMG828, and IncR plasmids. Resistance to multiple drugs compromises the treatment option for S. maltophilia limiting a potential treatment option that can cause serious nosocomial infections.

## Introduction

*Stenotrophomonas maltophilia* is an aerobic, non-glucose fermenting, Gram-negative bacillus that is frequently isolated from water, soil, animals, plant materials and hospital equipment. It was first isolated in 1943 as *Bacterium booker* and then named *Pseudomonas maltophilia* later, rRNA cistron analysis determined that it was more appropriately named Xanthomonas maltophilia [1]. Among the Gram-negative, non-fermenting bacteria, *S. maltophilia* causes most infections bar *Pseudomonas aeruginosa* and *Acinetobacter species*. It has emerged as an important nosocomial pathogen and can adhere to moist foreign surfaces and form biofilms, thus colonising the inanimate hospital environment and devices used for patient care [2]. *S. maltophilia* causes life-threatening infections among the vulnerable, and occasionally in immunocompetent individuals [3]. This bacterium is generally considered to be an opportunistic pathogen and can cause a range of infections, including bacteraemia, urinary tract infections, respiratory tract infections, skin and soft tissue infections, endocarditis, meningitis and ocular infections to mention but few. Although *S. maltophilia* causes mainly nosocomial infections, community-acquired infections have also been reported. It is inherently highly resistant to antibiotics and as a result infection is difficult to treat [3]. Infections caused by *S. maltophilia* have a high attributable mortality rate (37.5 %) [4], depending on the initial clinical condition of patients. Cross-infections between patients, transmitted by healthcare workers, have also been reported. Multiple mechanisms provide antibiotic resistance in *S. maltophilia*, including low membrane permeability, production of β-lactamases and aminoglycoside modifying enzymes, and, the presence of multidrug efflux pumps. In Nigeria, routine diagnostic microbiology does not isolate *S. maltophilia* and there is little information about the potential role of this organism in a country where antibiotic usage is accepted as being very high. This study was therefore embarked upon to isolate and characterise *S. maltophilia* from sick and healthy individuals in Nigeria.

## Methods

**Sample sites and bacterial isolates**: a total of 350 swab samples from various sites including; Toeweb, Nostril, Armpit, External ear and Antecubital fossa were collected from 70 healthy individuals living and working in Osogbo community as Medical students, Manual workers and Civil servants. These were compared with a clinical isolate collected from urine of a sick patient at Ladoke Akintola University of Technology Teaching Hospital (LTH). Ethical approval was obtained from the ethical committee, LAUTECH Teaching Hospital, Osogbo, Osun State. Individuals that participated in this study were duly informed and their consent was sought before they were recruited. *S. maltophilia* were isolated from overnight cultures on Blood and McConkey agar incubated at 37°C. Species assignments were confirmed for all isolates using cultural morphology, Gram reaction, standard biochemical tests and API 20 NE strips (BioMérieux, Basingstoke, UK) for Non-Enterobacteriaceae.

**Antibiotic susceptibility testing**: susceptibility of all isolates to a panel of antibiotic classes commonly used for treatment of *S. maltophilia* were determined by the agar dilution method on Mueller-Hinton agar according to the recommendations of CLSI breakpoints [5]. All runs included the control organism *Pseudomonas aeruginosa* (ATCC 27853) and *Escherichia coli* (ATCC 25922).

**Identification of ß-lactamase production**: the isolates were tested for production of extended spectrum β-lactams using the disc based ESBL detection set´ from Mast Group (Bootle, UK) and interpreted using the ‘ESBL detection set calculator’ tool as per the manufacturers' guidelines.

**Whole genome sequencing and bioinformatics**: total DNA was prepared from the isolates using Invisorb genomic mini prep kits. Successful preparation of DNA was confirmed using a Qubit instrument. Paired-end Illumina whole genome sequencing was completed using a NextSeq instrument at the Quadram Institute Bioscience, QIB. Bioinformatics used an inhouse pipeline hosted on an IRIDA instance; sequences were assembled with shovill and annotated with prokka and core snps identified with snippy. Core alignments were used to estimate phylogenetic relationships with IQ-Tree and trees visualized and annotated using the ‘interactive tree of life’ website. Furthermore, assemblies were used to search for plasmid content using the ‘PlasmidFinder’ tool hosted at the Centre for Genomic Epidemiology and for isolates likely to carry significant resistance genes in plasmids, ‘plasmidSPAdes’ was used to assemble likely plasmid contigs from the trimmed reads.

**Comparative genomics**: strains belonging to sequence types implicated in globally disseminated disease were compared against completed genomes where available to determine relationships between sources of strains, Nigerian strains and others in global circulation. Reads were also mapped against reference strains, SNPs identified and phylogenetic relationships determined as above to determine whether clones in Nigeria are divergent from those seen globally or highly similar suggestion transmission. Phylogenetic relationships identified in the datasets for each species were examined to identify closely related or identical strains or clusters of strains.

## Results

The 70 individuals who participated in the study comprised 33 Males and 37 Females with a mean age of 27 (range, 16-47). The occupation of the subjects was taken into consideration based on contact with the hospital environment, those who have had contact with the hospital environment (Medicine, Nursing, Medical Laboratory Science, Dietician), and those that do not have contact with the hospital environment. In total 2% of swabs (7/350) yielded a suspected *S. maltophilia* These were isolated from 5.7% (4/70) of healthy individuals (isolation of multiple strains of 2 to 3 from individuals), including a clinical *S. maltophilia* collected from urine of a sick patient at LTH (Table 1).

**Table 1:** demographic and Phenotypic Properties of *S. maltophilia*

WGS #	Lab ID	Site	Age	Sex	Specimen	ESBL	MICs (µg/ml)								
							LEV	CAZ	CHL	SMX	TMP	*TET	*AK	*KAN	*STR
ABC5	70Af	Carrier	29	F	Antecubital Fossa	+	4	64	>64	8	>64	64	>64	64	>64
ABC6	70N	Carrier	29	F	Nostril	-	2	64	>64	1024	>64	64	>64	64	>64
ABC10	70T	Carrier	29	F	Toeweb	+	32	>64	>64	1024	>64	64	>64	>64	>64
ABC11	69N	Carrier	32	M	Nostril	+	2	64	>64	1024	>64	64	>64	64	>64
ABC14	62N	Carrier	24	F	Nostril	+	4	>64	>64	8	64	64	>64	64	>64
ABC17	68Am	Carrier	23	M	Armpit	+	2	64	>64	64	>64	64	64	64	>64
ABC24	68Af	Carrier	23	M	Antecubital Fossa	+	0.5	64	>64	8	>64	64	64	64	>64
ABS3	121	Hospital	66	M	Urine	-	>64	>64	>64	1024	>64	64	>64	>64	>64

**Figure 1:**
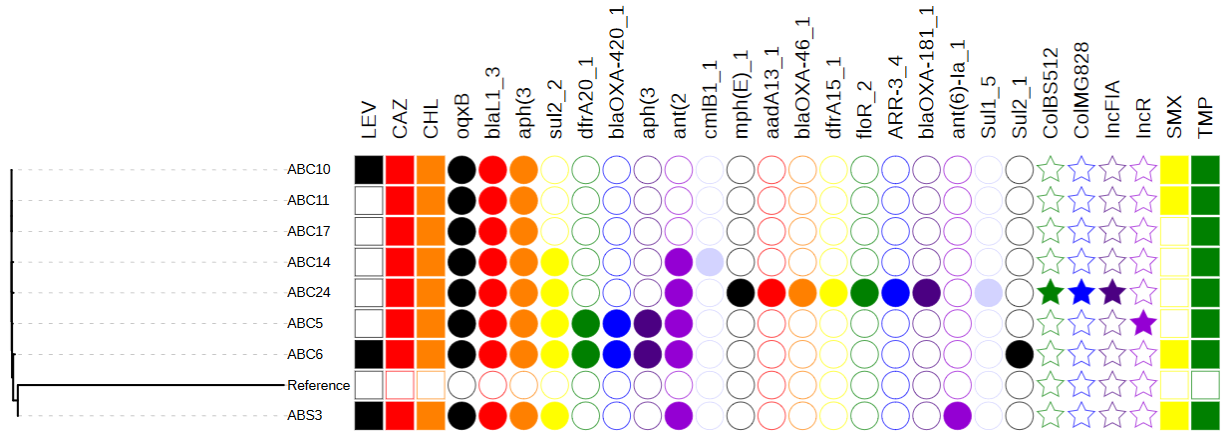
phylogenetic tree illustrating relatedness, resistance to antibiotics (presence of a filled square), antimicrobial resistance genes (represented by filled circles) and plasmid replicons (represented by filled stars); LEV: Levofloxacin; CAZ: Ceftazidime; CHL: Chloramphenicol; SMX: Sulphamethoxazole; TMP: Trimethoprim

Susceptibility results identified all isolates were resistant to all the four drugs which are part of the first-line drug policy with the exception of the few isolates that was susceptible to levofloxacin (MIC 0.5 to 4 µg/ml) and sulphamethoxazole (MIC 8 to 64 µg/ml). The other isolates had MIC50 and MIC90 values between 64 and >64 µg/ml, while for sulphamethoxazole MIC90 of the isolates was 1024 µg/ml. The only isolate from the sick was resistant to all the antibiotics (MIC 64 to 1024 µg/ml) (Table 1). Fluoroquinolone, beta-lactams and aminoglycosides resistance genes were found in all the isolates with the presence of oqxB, bla1_3 and aph (3) respectively. Other resistance genes present encoded resistance to sulphonamides (sul1, sul2), trimethoprim (dfrA15, dfrA20), florfenicol (floR), macrolides (mph(E)), and chloramphenicol (cmlB1) (Figure 1). Plasmid analysis only identified plasmid replicons from ABC5 and ABC24, in fact ABC24 had multiple plasmid replicons; ColBS512, ColMG828 and IncFIA, while ABC5 had an IncR replicon.

## Discussion

*S. maltophilia* has emerged as an important nosocomial pathogen and causes life-threatening infections among the vulnerable, and occasionally in immunocompetent individuals [3]. In this study a total of 2% of swabs (7/350) yielded a suspected *S. maltophilia* from 5.7% (4/70) of healthy individuals (isolation of multiple strains of 2 to 3 from individuals). This prevalence is consistent with other reports though these studies were from sick people [6]. All the 5.7% *S. maltophilia* were isolated from individuals with connections with the hospital, although it was unclear what the relationship of *S. maltophilia* and our local is. It is noteworthy *S. maltophilia* was also isolated from the urine of a sick person who is immunocompetent in our health facility.

The genotypes obtained correlates with the resistance patterns (phenotypes) of the isolates. This pattern of resistance (multiple drug resistance) results in treatment options that are extremely limited. It is obvious trimethoprim-sulphamethoxazole and other first-line drugs can no longer be drugs of choice except for levofloxacin (which apparently had its share of resistance), even high-level resistance was obtained for the isolate from the sick and found to be more resistant than the rest of the isolates (from healthy individuals). It is noteworthy that ABC5, ABC6 and ABC10 were isolated from the same individual, demonstrating carriage of more than one strain by individuals (Figure 1). This data suggests that most of the major resistance present in these isolates may be encoded on the chromosome although novel plasmids are in circulation in *S. maltophilia* and may act as vehicles to aid movement of AMR genes. IncR plasmids were first discovered in *Salmonella* encoding various *qnr* genes and have since been reported worldwide mainly in *K. pneumoniae* with various resistant genes including fluoroquinolones, aminoglycosides, carbapenems [7].

As far as we know, there has not been a report of infection by this agent in Nigeria and Africa since 1977 when Denis *et al*. [8] reported a case of meningitis in Africa when it was known as *Pseudomonas maltophilia*. This lack of information from Africa may be due to misdiagnosis of *S. maltophilia* with other bacterial isolates resulting in treatment failures from multiresistant strains since the organism is intrinsically resistant to multiple antibiotics including carbapenems.

## Conclusion

In conclusion, it was observed that carriage of *S. maltophilia* is related to occupation and that those with contact with the hospital environment have a higher percentage of carriage. The results indicate a high-level of multidrug resistance is common in *S. maltophilia* isolates from healthy carriers with resistance to the front-line treatments in all isolates. These results support a need for further surveillance of the prevalence, impact of *S. maltophilia* infection and its relationship with local environments in Nigeria as the resistance profile of isolates being carried suggests treatment will be difficult.

### What is known about this topic

- Stenotrophomonas maltophilia is generally considered to be an opportunistic pathogen and can cause a range of infections;
- It is inherently highly resistant to antibiotics and as a result infection is difficult to treat.

### What this study adds

- S. maltophilia was also isolated from the urine of a sick person who is immunocompetent in our health facility for the first time;
- Different strains (ABC5, ABC6 and ABC10) were isolated from the same healthy individual, demonstrating carriage of more than one strain by individuals;
- Trimethoprim-sulphamethoxazole and other first-line drugs can no longer be drugs of choice.
